# Satellite DNA Mapping in Suliformes (Aves): Insights into the Evolution of the Multiple Sex Chromosome System in *Sula* spp.

**DOI:** 10.3390/genes16060633

**Published:** 2025-05-24

**Authors:** Luciano Cesar Pozzobon, Natália dos Santos, Ricardo Utsunomia, Fábio Porto-Foresti, Marcelo de Bello Cioffi, Rafael Kretschmer, Thales Renato Ochotorena de Freitas

**Affiliations:** 1 Laboratório de Citogenética e Evolução, Departamento de Genética, Instituto de Biociências, Universidade Federal do Rio Grande do Sul, Porto Alegre 91509-900, RS, Brazil; lcpozzobon48@gmail.com (L.C.P.); thales.freitas@ufrgs.br (T.R.O.d.F.); 2 Faculdade de Ciências, Universidade Estadual de São Paulo, Bauru 17033-360, SP, Brazil; n.santos97@unesp.br (N.d.S.); ricardo.utsunomia@unesp.br (R.U.); fp.foresti@unesp.br (F.P.-F.); 3 Laboratório de Citogenética de Peixes, Departamento de Genética e Evolução, Universidade Federal de São Carlos, São Carlos 13565-905, SP, Brazil; mbcioffi@ufscar.br

**Keywords:** suliformes, satellite DNA, karyotypic evolution, multiple sex chromosomes, chromosomal mapping

## Abstract

Background: The order Suliformes exhibits significant karyotype diversity, with *Sula* species showing a Z_1_Z_1_Z_2_Z_2_/Z_1_Z_2_W multiple-sex chromosome system, an uncommon occurrence in avians. Satellite DNAs (satDNAs), which consist of tandemly repeated sequences, often vary considerably even among closely related species, making them valuable markers for studying karyotypic evolution, particularly that of sex chromosome evolution. This study aims to characterize and investigate the potential role of these sequences in the karyotypic evolution of the group, with special attention to the sex chromosomes. Methods: Through characterizing satDNAs in two Suliformes species (*Sula leucogaster* and *Nannopterum brasilianum*) using BGISEQ-500 platform and bioinformatics analysis. Their chromosomal distribution was mapped by fluorescence in situ hybridization (FISH) within their own karyotypes and in three additional Suliformes species (*S*. *sula*, *S*. *dactylatra*, and *Fregata magnificens*). Results: Five satDNAs were identified in *S. leucogaster* and eight in *N*. *brasilianum*. Within the genus *Sula*, three species shared specific satDNA sequences, although with different hybridization patterns. In contrast, the satDNAs of *N. brasilianum* were species-specific. Additionally, the Z chromosome, including Z_2_ in *Sula* species, showed reduced accumulation of repetitive DNAs. Conclusions: These results suggest that differential accumulation of repetitive sequences may have contributed to the diversification of karyotypes in this group, particularly influencing the structure and differentiation of sex chromosomes.

## 1. Introduction

Satellite DNA (satDNA) can constitute up to 50% of eukaryotic genomes, typically arranged in tandemly repeated monomers within the genome [1,2,3,4,5]. The term satellitome denotes the entire set of satDNAs within a species’ genome [6]. Characterizing the satellitome elucidates genome evolution through the examination of the presence and absence of specific satDNAs, their abundance, and their chromosomal distribution among various related species [7,8,9]. The Library Hypothesis, proposed by [10], affirms that satDNA can vary in copy number, frequency in the genome, and chromosomal localization among related species. The long-term conservation of satDNAs has been increasingly demonstrated across various groups, including plants [8], reptiles [11], nematodes [12], anurans [13], insects [9,14,15], fishes [16,17], and mammals [18,19,20]. In addition, [21] suggests that species that diverged over 50 million years ago (Mya) typically exhibit no similarities in their satellite DNAs (satDNAs).

Satellite DNAs (satDNAs), once considered “junk” due to their association with heterochromatin and low gene content [22], are now recognized as playing essential roles in genome function and organization. They are involved in cellular processes such as kinetochore assembly, X chromosome recognition, and meiotic segregation [23,24,25]. For instance, in *Drosophila melanogaster*, the 1.688 satDNA family is crucial for centromere function, heterochromatin formation, and dosage compensation [26,27]. Their rapid sequence evolution across species may contribute to hybrid incompatibilities and speciation [21,28,29].

In sex chromosomes, limited recombination results in independent evolution and accumulation of repetitive DNAs, particularly in W and Y chromosomes due to their haploid condition [7,30,31]. The W chromosome can harbor over 50% of repetitive DNAs in birds, whereas the entire genome comprises roughly 10% [32,33]. For example, in the Wattled Jacana *Jacana jacana,* four out of eleven satDNAs are present in the W chromosome, while none are found in the Z chromosome [34].

Thus far, the satellitome has been studied in a limited number of avian species, and, in contrast to other taxa, avian satDNAs are predominantly GC-rich and found in microchromosomes [34,35,36,37,38]. In *Turdus leucomelas*, two satDNAs were W-specific, TleSat06 and TleSat08, but a W-autosomal translocation with a small autosome, identified by comparative genomic hybridization, incorporated these satDNA sequences in a microchromosome [37].

In the order Suliformes, approximately 16% of species have undergone karyotyping, with the majority exhibiting diploid numbers (2n) lower than the hypothesized ancestral avian karyotype (2n = 80) [39,40,41,42,43,44,45,46,47]. The genus *Sula* also hosts rare cases of a multiple Z_1_Z_1_Z_2_Z_2_/Z_1_Z_2_W sex chromosome system in birds, where males display 2n = 76, characterized by Z_1_Z_1_Z_2_Z_2_ sex chromosomes, whereas females exhibit 2n = 75, possessing Z_1_Z_2_W sex chromosomes. A Robertsonian translocation between the W ancestral chromosome and a microchromosome was suggested as the most plausible hypothesis for the emergence of this system [47].

This study aims to investigate the role of the satellitome in the evolution of the karyotype of Suliformes using bioinformatics and molecular cytogenetic techniques, focusing on the sex chromosomes of the genus *Sula*. To achieve this, the satellitome of *S. leucogaster* and *N. brasilianum* was identified and subsequently hybridized into three closely related species (*S. dactylatra*, *S. sula,* and *F. magnificens*).

## 2. Materials and Methods

### 2.1. Sampling and Chromosomal Obtainment

The fibroblast cell culture was established using feather pulp from female and male individuals of *N. brasilianum, S. leucogaster*, *S. dactylatra*, *S. sula*, and *F. magnificens* (Table 1), following [48]. The individuals were captured in their natural environment and were promptly returned after sampling. Metaphase chromosome spreads were obtained by treatment with colchicine (0.05% at 37 °C) for 1 h, hypotonic solution (0.075 M KCl at 37 °C) for 8 min, and fixation with 3:1 methanol/acetic acid. Additionally, for *N. brasilianum* and *S. leucogaster*, 1 mL of blood was collected from the cutaneous ulnar vein for posterior DNA extraction. The sample collection was approved by the Sistema de Autorização e Informação em Biodiversidade (SISBIO) under protocol number 64234-5. Furthermore, the ethics committee from the Universidade Federal do Rio Grande do Sul (CEUA) approved the experiments involving animals under protocol number 42827.

### 2.2. DNA Extraction, Genome Sequencing, and Bioinformatic Analysis

The genomic DNA (gDNA) from one male and one female of *S. leucogaster* and *N. brasilianum* was extracted with the PureLink™ Genomic DNA Mini Kit (Invitrogen, Carlsbad, CA, USA), following the protocol provided by the manufacturer. Using the BGISEQ-500 platform at BGI (BGI Shenzhen Corporation, Shenzhen, China), these four libraries yielded 150 base pairs (bp) paired-end sequences, including 2 Gb for each of the female and male whole genomes.

Genomic libraries were quality-trimmed with Trimmomatic [49]. The satellitome was characterized by a female specimen of both *N. brasilianum* and *S. leucogaster*. Satellite DNA identification for each species was performed using multiple iterations of the TAREAN tool [50]. Initially, 2 × 500,000 reads were input into TAREAN to identify satellite sequences. The satDNAs detected were subsequently filtered using DeconSeq [51] until TAREAN no longer identified additional satDNA sequences. Other tandemly repeated sequences, such as multigene families, were also identified and removed. Next, a similarity search was conducted using RepeatMasker software 4.1.5 (https://github.com/fjruizruano/satminer/blob/master/rm_homology.py, accessed on 30 October 2023) to eliminate redundancies and categorize the identified satDNAs into three groups based on the classification proposed by [6]: (i) superfamilies (50–80% similarity), (ii) variants of a single satDNA family (80–95% similarity), and (iii) different copies of the same satDNA variant (more than 95% similarity). Using RepeatMasker [52], we calculated the divergence and abundance of each satDNA with a Python script (https://github.com/fjruizruano/ngs-protocols/blob/master/repeat_masker_run_big.py, accessed on 30 October 2023).

To estimate the relative abundance of each satDNA, we randomly selected 2 × 5,000,000 reads and mapped them against their satellitomes. The number of mapped reads was then divided by the number of analyzed nucleotides. The satDNA families were named incorporating the species abbreviation (Nbr for *N. brasilianum* and Sle for *S. leucogaster*), the term “Sat,” the catalog number, and the monomer size (in base pairs) in decreasing order of abundance. We also performed a comparative analysis between male and female specimens by using the satellites found and running RepeatMasker in males to find the differential abundance of satDNAs. A comparative analysis was also performed between female and male individuals to assess differential satDNA abundance. We considered a sex-exclusive satDNA when the presence was only in one sex or the ratio was <0.5 or >1.5. Additionally, we conducted a comparative analysis between *N. brasilianum* and *S. leucogaster* using the homology.v2 script (https://github.com/fjruizruano/satminer/blob/master/rm_homology.py, accessed on 25 April 2025) and de novo assembly in Geneious 7.1.3.

### 2.3. SatDNA Probes and Fluorescence In Situ Hybridization (FISH)

Primers were designed for three out of five described satDNAs for *S. leucogaster* and seven out of eight described satDNAs for *N. brasilianum* (Table S1). The satDNAs with the shorter monomer length for both species (SleSat03, SleSat04, and NbrSat07) were labeled with biotin at the 5′ end during synthesis by ThermoFisher (ThermoFisher Scientific). The satDNAs of both species were amplified by polymerase chain reaction (PCR) with a start denaturation at 95 °C for 5 min, followed by 35 cycles of denaturation at 95 °C for 45 s, annealing at 62–70.6 °C for 20–60 s, extension at 72 °C for 1 min, and final extension at 72 °C for 10 min (Table S2). The DNA concentrations used were 10 ng, 1 ng, 0.1 ng, 0.01 ng, 0.0001 ng, and 0.00001 ng. Ideal DNA template concentrations and amplification temperatures for each satDNA are described in Table S2. The PCR products were examined on a 2% electrophoresis gel and quantified in a NanoDrop LITE spectrophotometer (Thermo Scientific™, Wilmington, DE, USA) to ensure the amplification and integrity of satDNAs. The satDNAs amplified by PCR were labeled with the BioNick™ DNA Labeling System (Invitrogen, Carlsbad, CA, USA), following the manufacturer’s instructions.

The probes were hybridized following the protocol described by [53]. After hybridization, the probes were detected with Streptavidin-Cy3 dye, and the chromosomes were counterstained with 4′,6-diamidino-2-phenylindole (DAPI) solution.

### 2.4. Image Processing

The chromosomal spreads were analyzed in a Zeiss Axio Imager A2 (ZEISS, Oberkochen, Germany) microscope with a 64-bit AxioCam MRc (ZEISS) attached and processed using Axio Vision SE 64 REL 4.8.3 (ZEISS) software. At least 10 metaphase chromosomal spreads were analyzed to confirm the FISH results.

## 3. Results

The diploid number and chromosome morphologies of all five species in this study align with the previous description of their karyotypes [46,47]. *N. brasilianum* presented 2n = 74, *S. leucogaster* and *S. dactylatra* presented 2n = 75 for females and 2n = 76 for male individuals, *S*. *sula* presented 2n = 76 for males, and *F. magnificens* presented 2n = 76 (for both sexes).

### 3.1. Abundance and Characteristics of the satDNAs

Five satDNAs were identified in *S. leucogaster*, while *N. brasilianum* exhibited eight distinct satDNAs (Table 2). In *S. leucogaster*, the monomer lengths varied from 17 to 190 base pairs (bp), with three designated as long satDNAs, each surpassing 100 bp in length (SleSat01, SleSat02, and SleSat05). Conversely, *N. brasilianum* exhibited a broader diversity in monomer length, spanning from 20 to 2559 bp. Of these, four satDNA were categorized as short, each measuring less than 100 bp (NbrSat04, NbrSat04, NbrSat07, and NbrSat08), while the others were classed as lengthy (NbrSat01, NbrSat02, NbrSat05, and NbrSat06). NbrSat02 and NbrSat05 demonstrated lengths beyond 2 kilobases (Kb).

Differences between male and female libraries were observed for only one satDNA in each species (Figures S1 and S2, Table 2). In *S. leucogaster*, SleSat04 exhibited significantly higher abundance in the female compared to the male library, whereas in *N. brasilianum*, NbrSat07 was detected exclusively in the female library. No satDNA sequences were shared between the two species (*N. brasilianum* and *S. leucogaster*). Most satDNAs on the analyzed species showed a higher percentage of CG. In *S. leucogaster*, only SleSat04 deviated from this trend, displaying a lower C+G content (47.1%), while the other four ranged from 54.7% to 63%. In *N. brasilianum*, NbrSat04 (42.2%) and NbrSat06 (38.4%) showed lower C+G content, whereas the remaining ranged from 52.1% to 71.4%.

### 3.2. Chromosomal Location of S. leucogaster satDNAs (SleSatDNAs)

**Figure 1:**
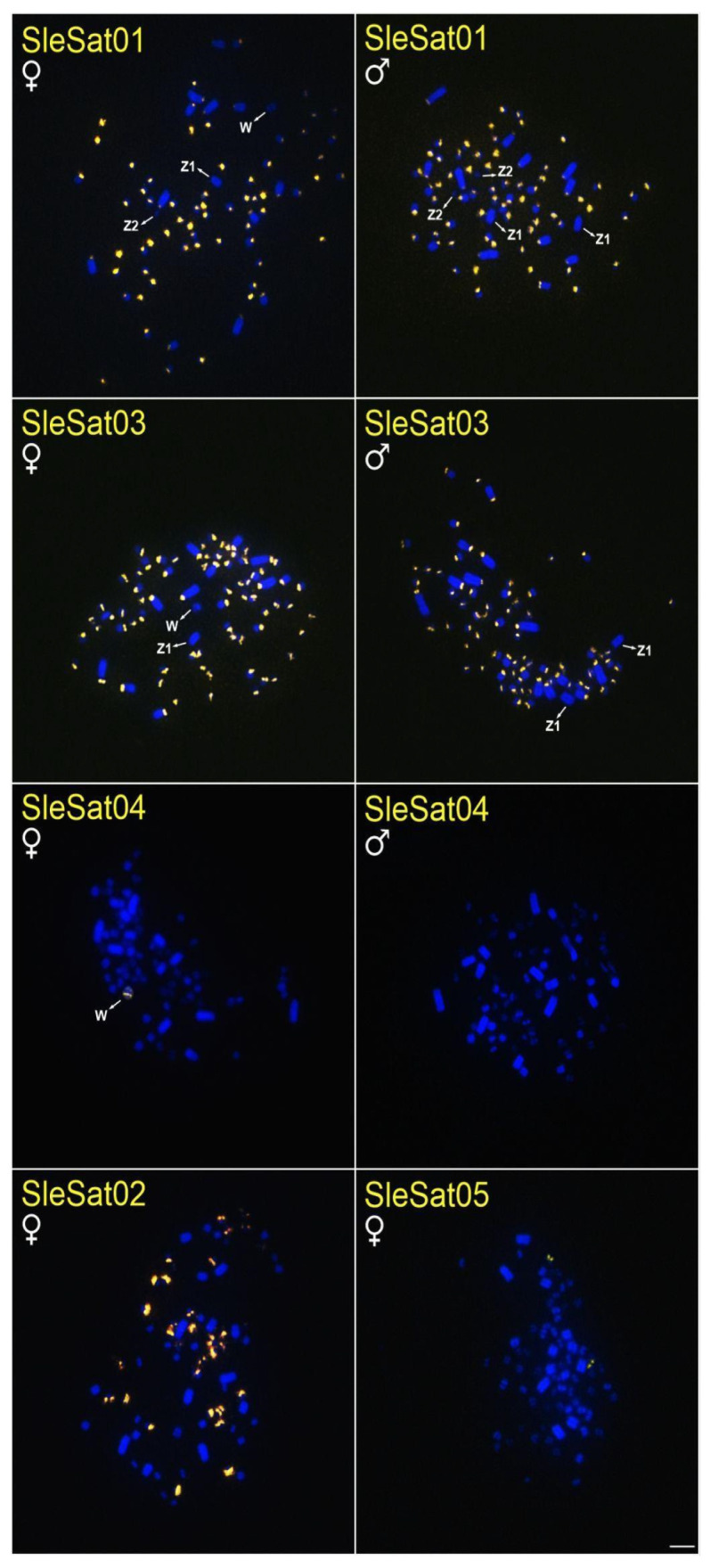
Metaphase plates of females and males of *S. leucogaster* hybridized with different SleSatDNAs. Bar = 5 µm.

**Figure 2:**
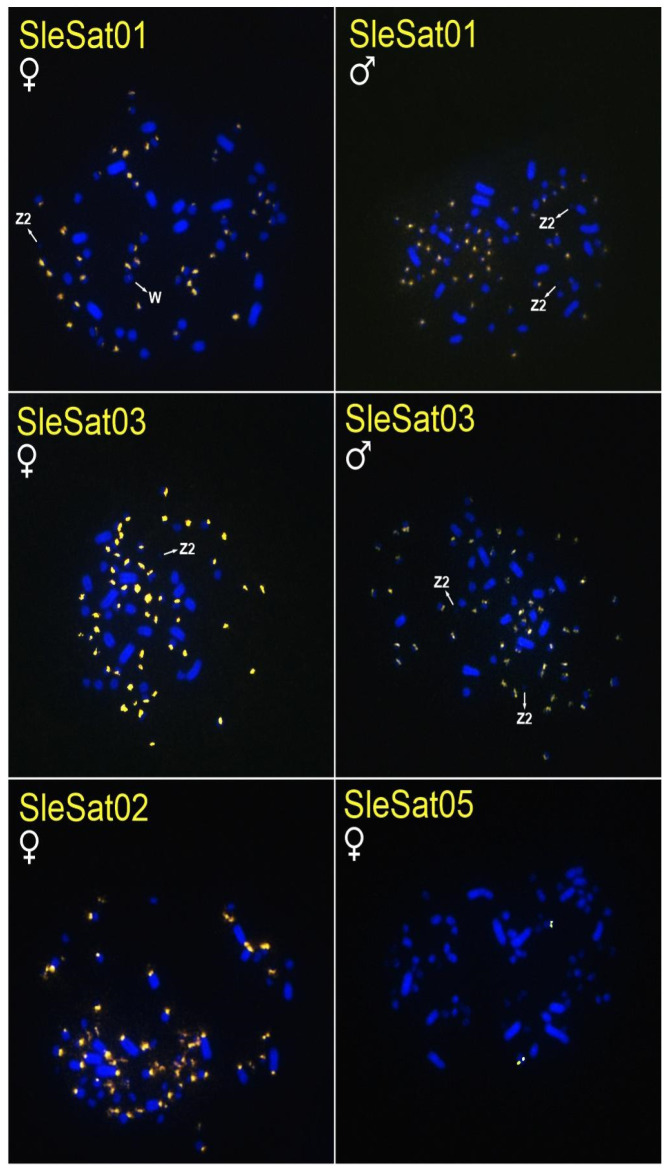
Metaphase plates of females and males of *S. dactylatra* hybridized with different SleSatDNAs. Bar = 5 µm.

**Figure 3:**
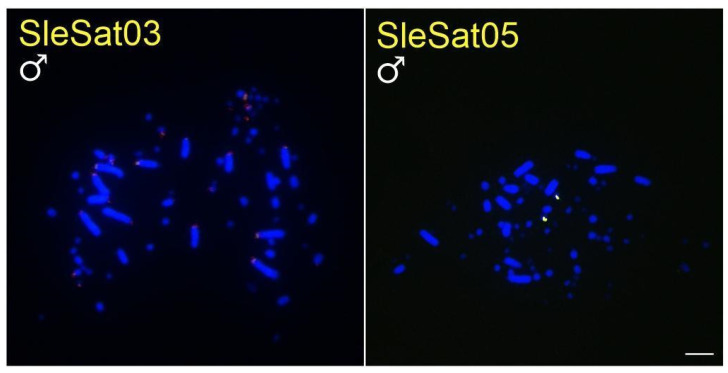
Metaphase plates of males of *S. sula* hybridized with different SleSatDNAs. Bar = 5 µm.

All SleSatDNAs exhibited distinct hybridization patterns on their chromosomes, with most hybridization signals detected in the microchromosomes (Table 3, Figure 1). Furthermore, all SleSatDNAs, except for SleSat04, exhibited hybridization signals on the chromosomes of *S. dactylatra* (Table 3, Figure 2), while only SleSat03 and SleSat05 were present on the chromosomes of *S. sula* (Table 3, Figure 3). It is important to highlight that there was no evidence of hybridization signals for SleSat01 on the sex chromosomes of *S. leucogaster* or SleSat03 on Z_2_ of *S. dactylatra*. Moreover, SleSat04 exclusively showed signs on the W chromosome of *S. leucogaster*. In contrast, SleSat05 exhibited the same hybridization pattern across all species within the genus *Sula*, with only one microchromosome pair showing hybridization signals.

SleSat01 probes produced no hybridization signals on the W chromosome of *S. leucogaster*, whereas weak signals were detected on the W chromosome of *S. dactylatra* (Table 3, Figure 1). Additionally, SleSat04, which is specific to the female library, exhibited hybridization signals exclusively on the W chromosomes of *S. leucogaster* (Table 3, Figure 1). Neither SleSat01 nor SleSat03 showed any signals on the Z chromosome; however, SleSat03 was detected on the Z_2_ chromosome of *S*. *leucogaster* (Table 3, Figure 1), and both SatDNAs failed to produce hybridization signals on the Z_2_ chromosome of *S. dactylatra* (Table 3, Figure 2).

The SleSatDNAs showed no hybridization signals in *N. brasilianum* and *F. magnificens* chromosomes, indicating the absence of these satDNAs in these species. It is important to consider the possibility of their existence in low abundance and/or scattered distribution, which would not be detected due to the limitations of the FISH technique.

### 3.3. Chromosomal Location of N. brasilianum satDNAs (NbrSatDNAs)

**Figure 4:**
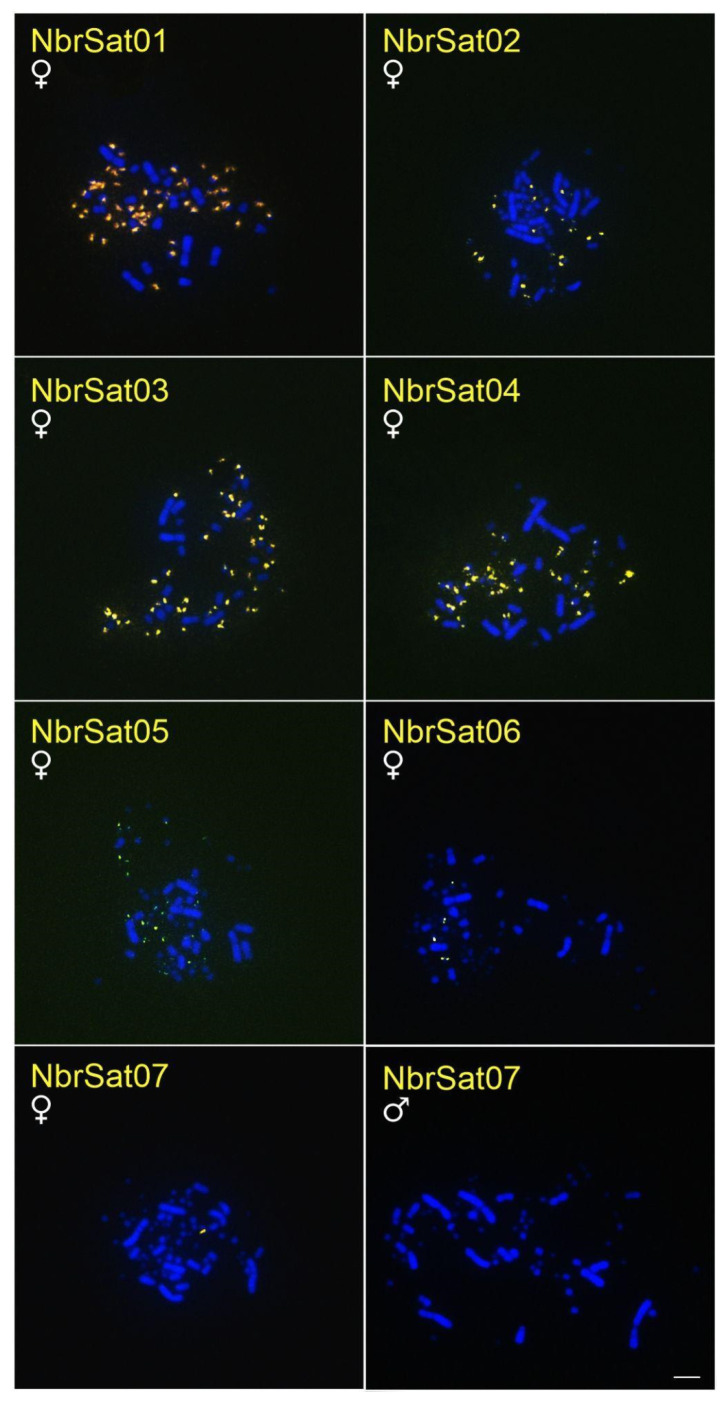
Metaphase plates of males and females of *N. brasilianum* hybridized with different NbrSatDNAs. Bar = 5 µm.

The NbrSatDNAs hybridization results revealed that, except for NbrSat07 and NbrSat08, all these sequences were localized on microchromosomes of *N. brasilianum* (Table 3, Figure 4). NbrSat01 and NbrSat03 had a similar hybridization pattern; however, NbrSat01 showed weaker signals (Table 3, Figure 4). NbrSat07 exclusively hybridized to the W sex chromosome (Table 3, Figure 4), whereas no signals were seen for NbrSat08. The Z sex chromosome showed no hybridization signals of any NbrSatDNAs (Table 3, Figure 4).

No FISH signals corresponding to any NbrSatDNAs were detected in *F. magnificens* and the three *Sula* species examined.

## 4. Discussion

In the present study, we characterized and compared, for the first time, the complete set of satellite DNAs (satellitome) in two Suliformes species, *S. leucogaster* and *N. brasilianum*. Additionally, we used fluorescence in situ hybridization (FISH) to investigate the presence of the identified satDNAs in three other species: *S. dactylatra*, *S. sula*, and *F. magnificens*. Our results revealed limited conservation of satDNA subsets across these species, with a higher number of sequences shared between closely related taxa, such as *S. leucogaster* and *S. dactylatra*. Furthermore, we demonstrated that the recently emerged Z_1_ and Z_2_ chromosomes in the genus *Sula* tend to accumulate fewer satDNA sequences compared to the autosomes and the W chromosome, a pattern consistent with previous findings for the typical Z chromosomes in birds.

### 4.1. Overview of Satellite DNA in S. leucogaster and N. brasilianum

We identified five satDNAs in *S. leucogaster* and eight in *N. brasilianum.* Among birds, the number of satDNA families ranges from four in *C. cristata* to twenty-eight in *Corvus woodfordi* and *C. splendens* [38,54]. The variety of satDNA numbers among various species is markedly variable, with a minimum of one in the moth *Cydalima perspectalis* and a maximum of 248 in the crayfish *Pontastacus leptodactylus* [14,55]. In closely related species, such as the root-knot nematode genus *Meloidogyne*, the amount of satDNAs may vary, ranging from 38 in *M. floridensis* to 81 in *M. arenaria* [12]. These differences in the number of satDNAs highlight the remarkable diversity of satDNA across species.

The AT content in bird satDNAs is typically lower compared to other taxa, a trend that extends to insects [56], amphibians [13], fishes [57], reptiles [58], and mammals [18,20]. The higher GC content in birds is widely documented [34,35,36,37,38,54,59,60] and may correlate with the comparatively GC-rich structure of their microchromosomes, as shown in *Gallus gallus* and *Meleagris gallopavo* [61,62]. Another potential reason is the pronounced preferential fixing of GC over AT in avian species [63].

DNA monomers surpassing 2 Kb in length have been observed in *N. brasilianum* and 12 other avian species, constituting around 37.5% of the 32 species examined, with the majority exhibiting one or two such monomers. The longest monomer was identified in the wattled *J. jacana,* reaching 6514 bp [34,35,36,37,38,54]. Longer satDNA sequences are variants of shorter ones that have undergone mutations [64]. The notably long satDNA monomers are extensively recorded in several species, indicating that they are a prevalent feature in birds as well [11,18,65,66,67].

### 4.2. Comparative Chromosomal Mapping of Satellite DNAs in Suliformes

Four satDNAs identified in *S. leucogaster* generated FISH signals in *S. dactylatra*, while only two were successfully hybridized in *S. sula* chromosomes. This pattern likely indicates the recent divergence of these species, with *S. sula* splitting from their common ancestor roughly 5.9 million years ago (Mya) and *S. leucogaster* and *S. dactylatra* diverging around 3.6 Mya [68]. The satDNAs of *N. brasilianum* hybridized only with its chromosomes. This pattern is likely due to its substantial divergence from the genus *Sula*, estimated at approximately 50 Mya [69]. Similarly, no evidence of hybridization signals was found in *F. magnificens*, which may be attributed to its divergence from the other four species around 59 Mya [69]. These results corroborate the findings of [21], who reported that plant and animal species with divergence times exceeding 50 Mya typically show no similarity in their repetitive DNA sequences. Furthermore, shared satDNA among the three *Sula* species supports the Library Hypothesis [10]. This hypothesis proposes that closely related species retain common satDNAs, though their genomic abundance may vary. The idea is exemplified by the five satDNAs of *S. leucogaster*, which persist within the genus *Sula* but differ in distribution.

Except for SleSat04 in *S. leucogaster* and NbrSat07 in *N. brasilianum*, all other satDNAs hybridized to centromeric regions and covered some microchromosomes (SleSat01, SleSat02, SleSat03, NbrSat01, NbrSat03, and NbrSat04). This pattern is similar to the satDNAs TleSat1 and TleSat3 of *T. leucomelas* and VchSat1 of *V. chilensis*, which also hybridized into centromeric regions [35,37]. According to [70], such distributions provide evidence that these satDNAs may have a significant role in the function of the centromere by either aiding the binding of proteins or serving mainly as structural components of the centromere.

### 4.3. Satellite DNA Mapping in Suliformes Sheds Light on Sex Chromosome Evolution

The absence of recombination in the W chromosome makes it highly susceptible to accumulating repetitive DNA [7]. Additionally, the size and repetitive DNA content of the W chromosome vary significantly among bird species [7,71,72]. In Suliformes, W morphology differs across all studied species [47]. This may result from the buildup of various repeating DNAs, including satellite DNA and transposable elements. Indeed, the SleSat04 hybridized exclusively to the W chromosome of *S. leucogaster*, with no signals detected in the other two *Sula* species (Figure 1). Similarly, NbrSat07 was restricted to the W chromosome of *N. brasilianum*, showing no hybridization signals in different species tested (Figure 4). This pattern of W-linked satDNA accumulation has been documented in five other avian species, where hybridization signals suggest a significant buildup of repetitive DNA on the W chromosome [34,35,36,37,38].

In most avian species, the Z chromosome shows little constitutive heterochromatin, mainly in the centromere region, though it may be entirely absent [41,60,73,74]. Corroborating these findings, previous studies have shown that the Z chromosome in *N*. *brasilianum* and the Z_1_ and Z_2_ chromosomes in the genus *Sula* lack constitutive heterochromatin [47]. In this study, we extend these findings by demonstrating that these chromosomes also exhibit few satDNA hybridization signals. This indicates that although satDNA exists on the Z chromosome, specifically SleSat03 on the Z_2_ chromosome of *S. leucogaster* (Figure 1), is comparatively scarce. Similar findings have been observed in *V. chilensis*, where only VchSat01 is located in the centromere of all chromosomes, including the Z chromosome [34]. Conversely, in the red-legged seriema *Cariama cristata,* the Z chromosome is the largest element in the karyotype, attributed in part to the accumulation of the satDNA CcrSat02-1104 [38]. Additionally, in the pale-breasted Thrush *T. leucomelas,* two satDNA sequences hybridize to the centromeric region of the Z chromosome, which is likewise heterochromatic [37].

## 5. Conclusions

Here, we describe the satDNA of *S. leucogaster* and *N. brasilianum*. Eleven of the thirteen satDNAs characterized have a higher content of GC, a general feature for bird satellites, since this observation was also made in other birds. Also, most satDNA were localized in the same sites as constitutive heterochromatin and centromere sites. Ref. [21] stated that species that diverged more than 50 Mya showed no satDNA resemblance. This statement is confirmed here since only the genus *Sula,* with 5.9 Mya of divergence, showed shared satDNAs [69]. Finally, the Z chromosome of *N. brasilianum* and *S. leucogaster* showed none to little satDNA hybridization signals, indicating that the Z chromosome has little tendency to accumulate satDNA, including the Z_2_ of genus *Sula*.

## Figures and Tables

**Table 1:** The locality and family of the five species of the Suliformes order analyzed in this study.

Species	Family	Sex	Locality
*N. brasilianum*	Phalacrocoracidae	1♀1♂	Rio Grande, RS, Brazil
*S. leucogaster*	Sulidae	1♀1♂	Abrolhos, PE, Brazil
*S. dactylatra*	Sulidae	1♀1♂	Abrolhos, PE, Brazil
*S. sula*	Sulidae	1♂	Fernando de Noronha, BA, Brazil
*F. magnificens*	Fregatidae	1♀	Abrolhos, PE, Brazil

**Table 2:** General characteristics of satDNAs found in *S. leucogaster* (SleSat) and *N. brasilianum* (NbrSat).

Satellite	Monomer Size	Abundance (F)	Abundance (M)	Ratio F/M	C + G (%)	A + T (%)
SleSat01	100	0.114238948	0.103363981	1.105210408	63	37
SleSat02	190	0.044193639	0.041334463	1.069171739	54.7	45.3
SleSat03	24	0.016987825	0.014847279	1.144170977	62.5	37.5
SleSat04	17	0.004358942	0.0000005813	7498.180046	47.1	52.9
SleSat05	145	0.000124061	0.000156155	0.794473001	55.2	44.8
NbrSat01	113	0.016387741	0.019163133	0.855170235	66.4	33.6
NbrSat02	2559	0.004029267	0.004085564	0.986220589	52.1	47.9
NbrSat03	42	0.002012025	0.002329438	0.863738521	71.4	28.6
NbrSat04	62	0.000820984	0.000918009	0.89430964	42.2	57.8
NbrSat05	2118	0.000731359	0.000866913	0.843635922	61.7	38.3
NbrSat06	862	0.000214127	0.000206287	1.03800201	38.4	61.6
NbrSat07	20	0.000155798	-	-	60	40
NbrSat08	77	0.0000995020	0.000116493	0.854148187	68.8	31.2

F = female; M = male; C + G = cytosine and guanine; A + T = adenine and thymine.

**Table 3:** Hybridization results of SatDNA families found in *S. leucogaster* (SleSat) and *N. brasilianum* (NbrSat) and in three closely related species (*S. dactylatra*, *S. sula*, and *F. magnificens*).

Satellite	*S. leucogaster*	*S. dactylatra*	*S. sula*	*N. brasilianum*	*F. magnificens*
SleSat01	centromeres of autosomes	46 microchromosomes and the centromere of W	no signal	no signal	no signal
SleSat02	34 microchromosomes	20 macrochromosomes and 34 microchromosomes	no signal	no signal	no signal
SleSat03	centromeres of autosomes and Z2	52 microchromosomes	4 microchromosomes and the first 8 pairs of macrochromosomes	no signal	no signal
SleSat04	chromosome W	no signal	no signal	no signal	no signal
SleSat05	2 microchromosomes	2 microchromosomes	2 microchromosomes	no signal	no signal
NbrSat01	no signal	no signal	no signal	52 microchromosomes	no signal
NbrSat02	no signal	no signal	no signal	16 microchromosomes	no signal
NbrSat03	no signal	no signal	no signal	52 microchromosomes	no signal
NbrSat04	no signal	no signal	no signal	24 microchromosomes	no signal
NbrSat05	no signal	no signal	no signal	16 microchromosomes	no signal
NbrSat06	no signal	no signal	no signal	4 microchromosomes	no signal
NbrSat07	no signal	no signal	no signal	W chromosome	no signal
NbrSat08	no signal	no signal	no signal	no signal	no signal

## Data Availability

The sequencing reads have been deposited in the Sequence Read Archive (SRA-NCBI) and are available under the following accession numbers: *Nannopterum brasialianum*—SRR33660189 (male) and SRR33660190 (female); *Sula leucogaster*—SRR33660187 (male) and SRR33660188 (female). The original contributions presented in this study are included in the article/Supplementary Material.

## Supplementary Materials

The following supporting information can be downloaded at https://www.mdpi.com/article/10.3390/genes16060633/s1, Table S1: Primers design for amplification of satellitomes from *Sula leucogaster* (SleSat) and *Nannopterum brasialianum* (NbrSat); Table S2: Termocycle program for amplification of satellitomes from *Sula leucogaster* (SleSat) and *Nannopterum brasialianum* (NbrSat); Figure S1: Landscape from female (A) and male (B) of *Nannopterum brasilianum* with satDNA families in order of abundance; Figure S2: Landscape from female (A) and male (B) of *Sula leucogaster* with satDNA families in order of abundance.
