# Supplementary material for: Satellite DNA Mapping in Suliformes (Aves): Insights into the Evolution of the Multiple Sex Chromosome System in Sula spp
Source: Genes (Basel). 2025 May 24;16(6):633. doi: 10.3390/genes16060633 (PMC12193320; doi:10.3390/genes16060633)
Supplement: Supplementary file 1 [file genes-16-00633-s001.zip › genes-3663383-supplementary.pdf]

SUPPLEMENTARY INFORMATION

Supplemantary Table 1. Primers desing for amplification of Satellitomes from *Sula leucogaster* (SleSat) and *Nannopterum brasialianum* (NbrSat)

Satellite	Forward	Reverse
SleSat01	AGCAGCGTGCATGGGACTG	CCATGCAACGCTGCCCCGT
SleSat02	GTGGCTGTGAAAGGGTTGTC	ACCAACACTGGGCCTCTG
SleSat03	Biotinylated Oligonucleotide	
SleSat04	Biotinylated Oligonucleotide	
SleSat05	CGAGACTCCTCCCAGGCA	GGCAGATCCCAGGGCAG
NbrSat01	AACCGGACAGGATCGGGCC	TTTGCACGGCAGTCCGTTCC
NbrSat02	CAGTGCCTCACAGTACGTCAAC	AGGGAGCCACTCAGAGCGA
NbrSat03	CCATTCCGGGGGCCGGAT	AATGGACTGCCGCGGAAAAC
NbrSat04	AAGCCTCATTTTTAGGACCCAA	TGAGCGTTAAAAGAGGGGTTTG
NbrSat05	CTGAGACTCCCGCAGCACG	TCTCAGTCCACCCATCCTTCC
NbrSat06	TTTGCCTTATTTCCTTCACCC	CGGAAAGACATTTTGGAAAGCG
NbrSat07	Biotinylated Oligonucleotide	
NbrSat08	CTCTCTGCTGCCACCGTGC	CAGAAAAGGGCGCCACTGC

Supplementary Table 2. Termocycle program for amplification of Satellitomes from *Sula leucogaster* (SleSat) and *Nannopterum brasialianum* (NbrSat)

Satellite	Desnaturation	Cycles	Desnaturation	Annealing	Amplification	Final amplification	Concentration (ng/ul)
SleSat01	95 ºC for 5 min	35	95 ºC for 45 s	70.6 ºC for 20 s	72 ºC for 1 min	72ºC for 10 min	0,00001
SleSat02	95 ºC for 5 min	35	95 ºC for 45 s	61 ºC for 1 min	72 ºC for 1 min	72ºC for 10 min	0,0001
SleSat03	Biotinylated Oligonucleotide						
SleSat04	Biotinylated Oligonucleotide						
SleSat05	95 ºC for 5 min	35	95 ºC for 45 s	61.7 ºC for 40 s	72 ºC for 1 min	72ºC for 10 min	0,01
NbrSat01	95 ºC for 5 min	35	95 ºC for 45 s	69 ºC for 20 s	72 ºC for 1 min	72ºC for 10 min	0,0001
NbrSat02	95 ºC for 5 min	35	95 ºC for 45 s	64 ºC for 1 min	72 ºC for 1 min	72ºC for 10 min	0,1
NbrSat03	95 ºC for 5 min	35	95 ºC for 45 s	69 ºC for 40 s	72 ºC for 1 min	72ºC for 10 min	0,01
NbrSat04	95 ºC for 5 min	35	95 ºC for 45 s	64 ºC for 40 s	72 ºC for 1 min	72ºC for 10 min	10
NbrSat05	95 ºC for 5 min	35	95 ºC for 45 s	64 ºC for 1 min	72 ºC for 1 min	72ºC for 10 min	1
NbrSat06	95 ºC for 5 min	35	95 ºC for 45 s	62 ºC for 1 min	72 ºC for 1 min	72ºC for 10 min	1
NbrSat07	Biotinylated Oligonucleotide						
NbrSat08	95 ºC for 5 min	35	95 ºC for 45 s	68 ºC for 1 min	72 ºC for 1 min	72ºC for 10 min	0,1

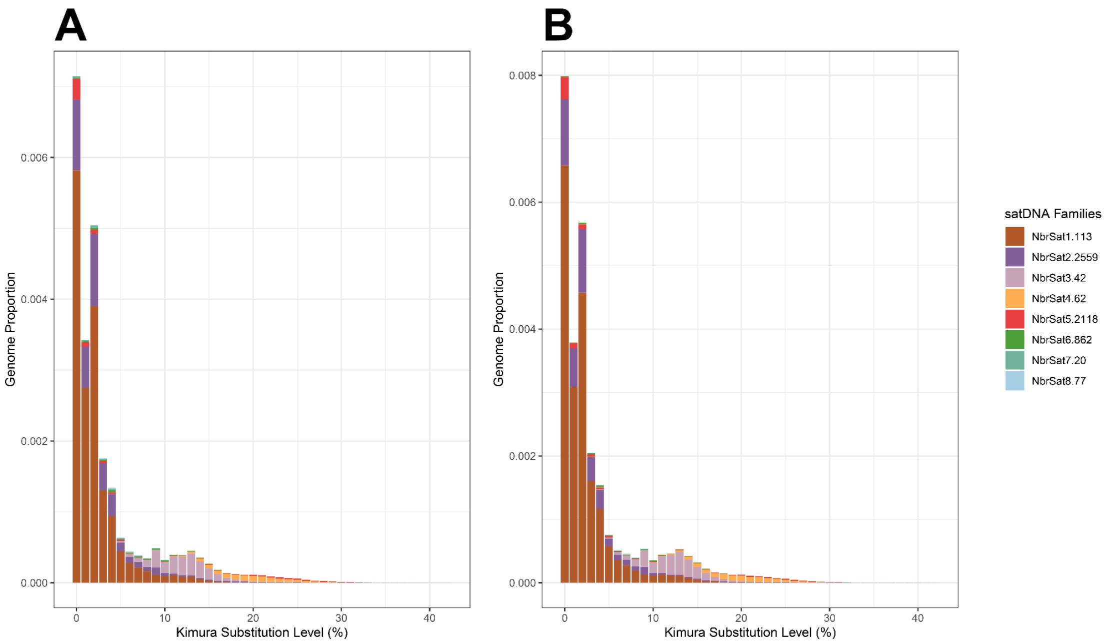

Supplementary Figure 1. Landscape from female (A) and male (B) of *Nannopterum brasilianum* with satDNA families in order of abundance.

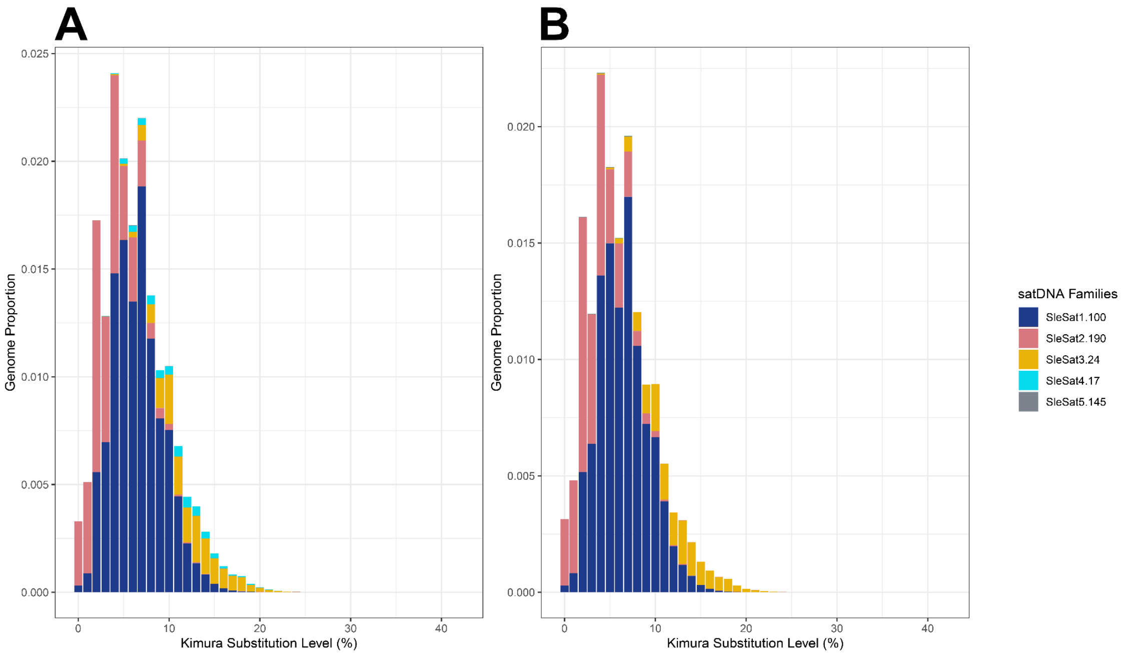

Supplementary Figure 2. Landscape from female (A) and male (B) of *Sula leucogaster* with satDNA families in order of abundance.
